# Awakening the Vagus: Transcutaneous Auricular Vagus Nerve Stimulation—A Narrative Review

**DOI:** 10.3390/brainsci16090979

**Published:** 2026-09-16

**Authors:** Lara Portincaso, Gaia Chiara Santi, Nicole Villa, Davide Antonio Di Pietro

**Affiliations:** 1 Istituti Clinici Zucchi, Gruppo San Donato, 20841 Carate Brianza, Monza Brianza, Italy; lara.portincaso@gmail.com; 2 Neurorehabilitation Unit of the Institute of Lumezzane, Istituti Clinici Scientifici Maugeri IRCCS, 25065 Lumezzane, Brescia, Italy; gaia.santi@icsmaugeri.it (G.C.S.); nicole.villa@icsmaugeri.it (N.V.)

**Keywords:** ascending reticular activating system, auricular vagus nerve stimulation, disorders of consciousness, minimally conscious state, neuromodulation, non-invasive brain stimulation, thalamocortical connectivity, unresponsive wakefulness syndrome

## Abstract

**Highlights:**

**What are the main findings?**

- taVNS has a potential neuromodulatory effect that can influence cortical and EEG reactivity, particularly in patients in a minimally conscious state.

**What are the implications of the main findings?**

- taVNS is a low-risk, easily implementable adjunct for a vulnerable population with few treatment options.
- Larger multicenter RCTs with standardized protocols are needed before considering routine clinical adoption of taVNS.

**Abstract:**

Disorders of consciousness (DoC), including unresponsive wakefulness syndrome/vegetative state (UWS/VS) and minimally conscious state (MCS), remain a major challenge in clinical neurology. Therapeutic options are limited, and prognosis is often uncertain. Transcutaneous stimulation of the auricular branch of the vagus nerve (taVNS) is a non-invasive neuromodulation technique that has gained increasing interest as a potential strategy to promote recovery of consciousness. This narrative review summarizes the anatomical and functional rationale of taVNS, compares invasive and non-invasive vagus nerve stimulation techniques, reviews the biological mechanisms underlying its effects, discusses established clinical applications, technological innovations, and available clinical trial evidence. Current data suggest that taVNS is safe and well-tolerated, with preliminary evidence of applicability in DoC, particularly in patients with MCS. However, heterogeneity in stimulation protocols and the limited number of large-scale randomized controlled trials highlight the need for further standardized research.

## 1. Introduction

Disorders of consciousness (DoC) arise from severe acquired brain injury and are characterized by impaired arousal and awareness [1,2]. Despite advances in diagnostic assessment, effective therapeutic strategies remain scarce. In recent years, neuromodulation techniques have emerged as promising approaches to enhance residual brain network activity and facilitate recovery of consciousness.

The vagus nerve plays a pivotal role in regulating arousal, autonomic balance, and thalamocortical connectivity through extensive afferent projections to the brainstem and forebrain [3]. Transcutaneous auricular vagus nerve stimulation (taVNS) exploits the auricular branch of the vagus nerve to modulate these pathways non-invasively, avoiding the risks associated with surgical implantation [4,5]. This review provides a comprehensive overview of the anatomical rationale, mechanisms of action, technological developments, and clinical evidence supporting taVNS in different medical conditions (i.e., psychiatric, neurological, and systemic disorders) and specifically in disorders of consciousness.

## 2. Methods

This narrative review was based on searches conducted in the MEDLINE/PubMed, Scopus, Web of Science, and Cochrane Library databases, supplemented by manual citation tracking (backward and forward) of key articles, given the limited number of taVNS studies specific to disorders of consciousness (DoC). Searches regarding fundamental anatomical and physiological literature on the vagus nerve were not subject to time limits, whereas clinical and experimental studies specific to taVNS covered the period from 2013 to June 2026. Searches were restricted to English-language publications.

Search strings combined MeSH/Emtree terms and free-text keywords related to the stimulation technique with terms concerning disorders of consciousness: (“transcutaneous auricular vagus nerve stimulation” OR “taVNS” OR “tVNS” OR “transcutaneous vagus nerve stimulation” OR “noninvasive vagus nerve stimulation”) AND (“disorders of consciousness” OR “unresponsive wakefulness syndrome” OR “vegetative state” OR “minimally conscious state” OR “prolonged disorders of consciousness” OR “coma”). Additional terms (“epilepsy”, “depression”, “migraine/headache”, “stroke”, “atrial fibrillation”, “chronic pain”, “autonomic nervous system”, “heart rate variability”, “stimulation parameters”, “safety/adverse effects”) were combined with terms related to vagal stimulation to include literature on the mechanisms and clinical applications relevant to the review.

The included studies comprised: randomized controlled trials, pilot/feasibility studies, case reports/case series, registered study protocols, systematic reviews/meta-analyses, relevant clinical guidelines, and preclinical/mechanistic studies supporting the neurobiological rationale. The following were excluded: conference abstracts lacking a full text, non-peer-reviewed sources, and non-English articles. Title and abstract screening and full-text assessment were performed by LP and DADP, with any disagreements resolved by DADP.

## 3. Anatomical and Functional Rationale

### 3.1. The Parasympathetic Nervous System

The parasympathetic nervous system (PNS) constitutes the craniosacral division of the autonomic nervous system and plays a central role in regulating visceral homeostasis, cardiovascular and respiratory control, gastrointestinal motility and secretion, immune modulation, and neuroendocrine balance [6]. Beyond its classical “rest-and-digest” function, contemporary neurobiological models conceptualize the PNS as an integral component of distributed brain–body networks contributing to arousal regulation, interoceptive processing, emotional modulation, and adaptive behavior [7,8].

Parasympathetic preganglionic neurons originate in discrete brainstem nuclei associated with cranial nerves III (Edinger–Westphal nucleus), VII (superior salivatory nucleus), IX (inferior salivatory nucleus), and X (dorsal motor nucleus of the vagus and nucleus ambiguus), as well as in the sacral spinal cord (S2–S4) [6]. These neurons project to peripheral ganglia located near or within target organs, enabling fine-grained, organ-specific modulation. Among all parasympathetic pathways, the vagus nerve (cranial nerve X) represents the dominant anatomical and functional connection between the central nervous system and the viscera [3].

### 3.2. Structural and Functional Organization of the Vagus Nerve: Afferent and Efferent Pathways

The vagus nerve is a mixed nerve composed of both sensory (afferent) and motor (efferent) fibers, with a marked predominance of afferent components. Approximately 70–80% of vagal fibers are indeed sensory, conveying visceral information from thoracic and abdominal organs to the central nervous system [7,8]. These afferent fibers arise from mechanoreceptors, chemoreceptors, baroreceptors, and nociceptors located in the heart, lungs, gastrointestinal tract, liver, and other visceral structures. The cell bodies of vagal afferent neurons are primarily located in the inferior (nodose) ganglion, with a smaller contribution from the superior (jugular) ganglion [7].

**Figure 1:**
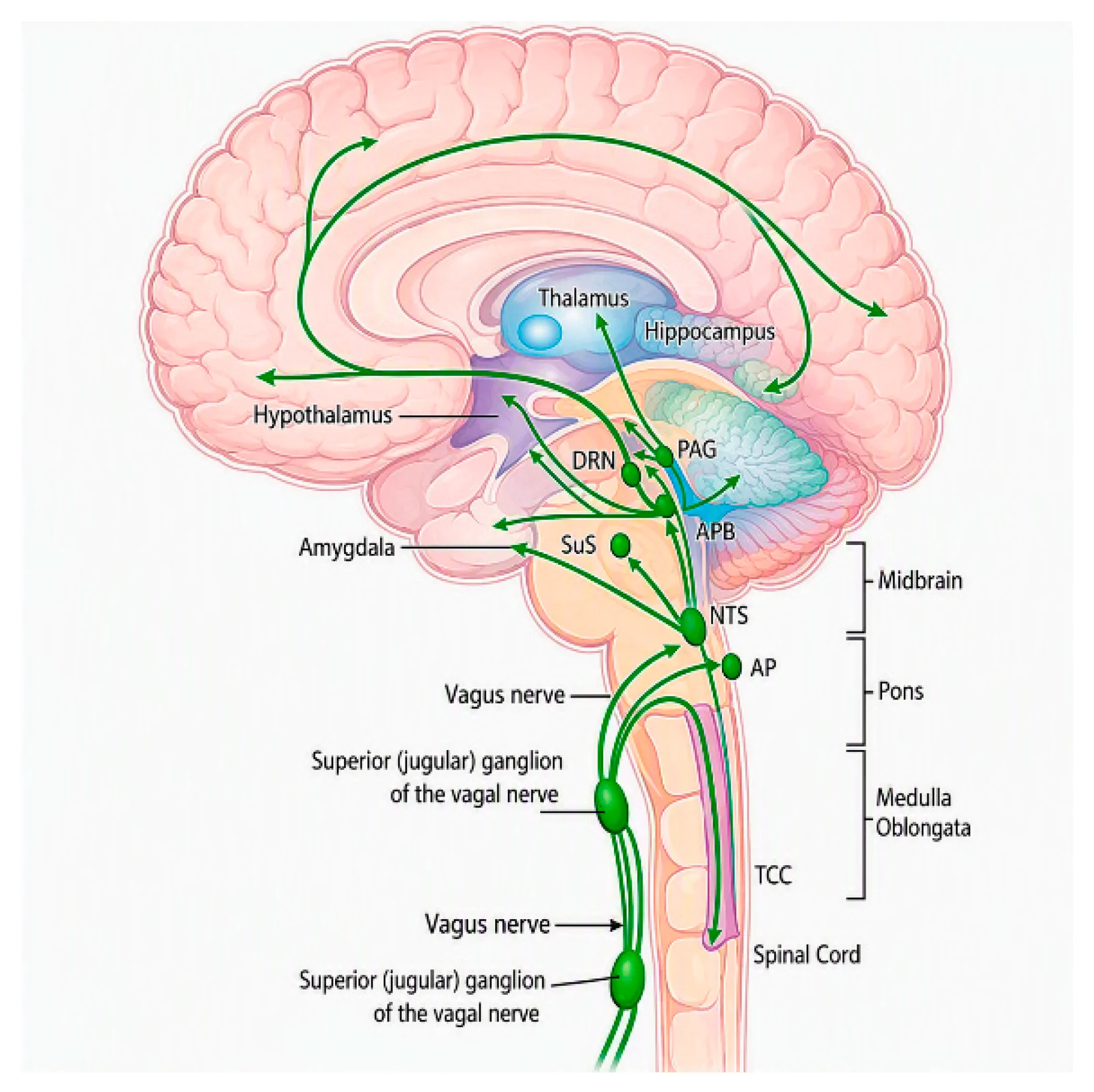
Central projections of the NTS and their relevance for vagus nerve stimulation. Schematic representation of vagal afferent pathways from the jugular and nodose ganglia to the NTS and their ascending projections to brainstem nuclei, thalamus, limbic structures, and cerebral cortex. Abbreviations: AP, area postrema; PAG, periaqueductal gray; PB, parabrachial nucleus; DRN, dorsal raphe nucleus; LC, locus coeruleus; NTS, nucleus tractus solitarius; SuS, superior salivatory nucleus (preganglionic parasympathetic neurons); TCC, trigeminocervical complex (trigeminal nucleus caudalis and its cervical extension to C1–C2).

Central projections of vagal afferents terminate predominantly in the nucleus tractus solitarius (NTS), located in the dorsomedial medulla oblongata [3,7]. The NTS serves as the principal integrative hub for visceral sensory input and plays a pivotal role in integrating autonomic reflexes, including cardiovascular, respiratory, and gastrointestinal regulation. Importantly, the NTS functions not merely as a relay nucleus but as a highly integrative structure that interfaces visceral afferent signals with central neuromodulatory systems (Figure 1) [7,8].

From the NTS, second-order projections extend to multiple brainstem and forebrain regions, including the locus coeruleus, dorsal raphe nuclei, parabrachial nucleus, hypothalamus, thalamus, amygdala, hippocampus, insular cortex, and prefrontal cortex [9,10,11]. Through these ascending pathways, vagal afferent signaling modulates noradrenergic and serotonergic neurotransmission, thalamocortical excitability, and large-scale brain network dynamics relevant to arousal, attention, and conscious awareness [9,10].

Vagal efferent fibers originate primarily from the dorsal motor nucleus of the vagus (DMNV) and the nucleus ambiguus [3]. The DMNV provides preganglionic parasympathetic innervation to abdominal and thoracic viscera [3,11]. The nucleus ambiguus also gives rise to cardioinhibitory fibers modulating heart rate and atrioventricular conduction [3].

Beyond classical autonomic control, vagal efferent activity may also contribute to immune regulation via the cholinergic anti-inflammatory pathway. Through acetylcholine-mediated interactions with immune cells, vagal efferents modulate cytokine release and systemic inflammatory responses, thereby linking autonomic output to immune homeostasis [12,13,14].

## 4. Central Integration and Therapeutic Implications

The anatomical organization of the vagus nerve places it at a strategic interface between peripheral physiology and central neural processing. Through its extensive afferent projections to the nucleus tractus solitarius (NTS) and the subsequent recruitment of neuromodulatory brainstem nuclei, the vagus nerve exerts a significant influence over the ascending reticular activating system and thalamocortical circuits [9,10]. These interconnected systems are critically involved in maintaining wakefulness, regulating vigilance, and supporting conscious awareness [15]. By modulating noradrenergic and serotonergic tone and influencing thalamocortical excitability, vagal afferent signaling can shape large-scale network dynamics and promote functional integration across distributed cortical and subcortical regions [9,10,15].

**Figure 2:**
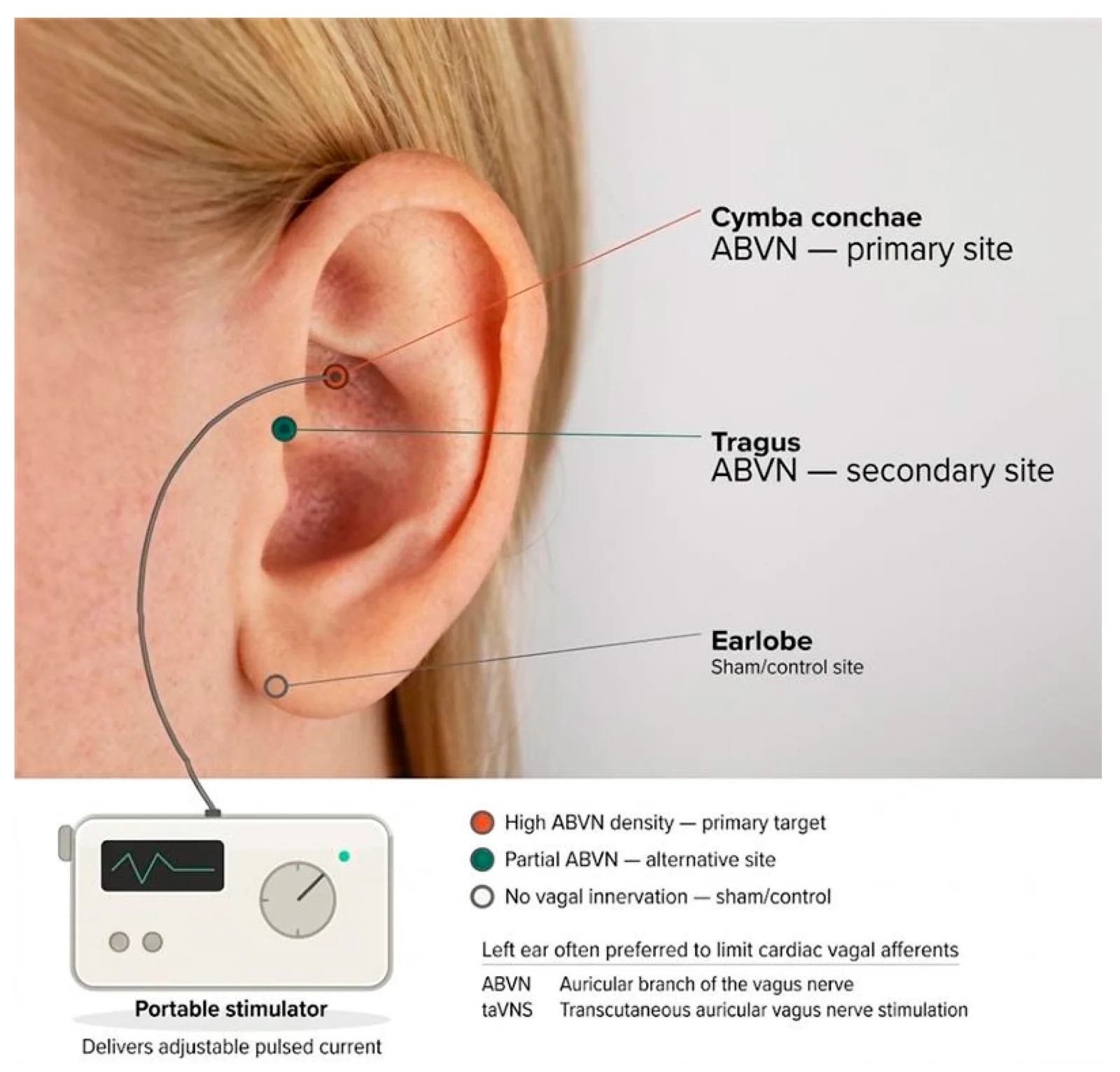
Electrode sites for transcutaneous auricular vagus nerve stimulation (taVNS): cymba conchae (primary target), tragus (alternative site), and earlobe (sham/control site, lacking vagal innervation), shown with the portable stimulator device. ABVN, auricular branch of the vagus nerve.

Building on this neuroanatomical and neurophysiological framework, vagus nerve stimulation is a mechanism-driven strategy that indirectly engages central arousal systems without requiring direct cortical intervention. In this context, transcutaneous auricular vagus nerve stimulation (taVNS) has emerged as a particularly promising approach. taVNS targets the auricular branch of the vagus nerve, which innervates specific regions of the external ear, most notably the cymba conchae (Figure 2) [4,16]. Afferent fibers from this branch project to the NTS, thereby providing non-invasive access to central vagal pathways while largely sparing efferent fibers involved in cardiovascular and gastrointestinal regulation [4].

This preferential activation of afferent projections contributes to the technique’s favorable safety and tolerability profile while preserving its capacity to modulate core neuromodulatory circuits.

Within this conceptual framework, the relevance of vagal neuromodulation becomes particularly evident in disorders characterized by disruption of ascending arousal pathways, impaired thalamocortical connectivity, and reduced integration across large-scale brain networks. In disorders of consciousness, where residual neural networks may persist but remain functionally disconnected, modulation of vagal afferent signaling offers a biologically plausible means of enhancing central arousal and facilitating network reintegration, thereby providing a coherent neurobiological rationale for the application of taVNS in this clinical population [15].

### Clinical Experience with taVNS Beyond Disorders of Consciousness

Clinical experience across neurological, psychiatric, and systemic conditions provides preliminary support for the therapeutic and neuromodulatory potential of taVNS (Table 1). Building on the established efficacy of implantable vagus nerve stimulation in drug-resistant epilepsy, early randomized and pilot studies of taVNS have reported reductions in seizure frequency and improvements in quality of life, although its effects appear less consistent and generally more modest than those achieved with invasive stimulation [17,18,19]. In major depressive disorder, RCTs have shown reductions in depressive symptoms compared with sham stimulation, while neuroimaging findings suggest modulation of limbic and prefrontal regions and changes in the functional connectivity of the default mode and salience networks [20,21,22,23]. Studies in migraine have similarly reported reductions in attack frequency, pain intensity, and medication use, potentially reflecting modulation of trigeminovascular pathways and descending pain-control systems [24,25]. Beyond symptom-specific effects, findings from autonomic and neurorehabilitation research may be particularly relevant to disorders of consciousness. Studies conducted in cardiovascular populations have associated taVNS with increased heart rate variability, reduced sympathetic activity, and improved baroreflex sensitivity, suggesting a potential influence on sympathovagal balance [26,27]. In stroke rehabilitation, vagal stimulation paired with motor training has been associated with greater functional improvement than rehabilitation alone, supporting the hypothesis that vagal afferent activation may facilitate activity-dependent plasticity through neuromodulator release and synaptic strengthening [28,29]. Collectively, these findings indicate that taVNS can engage neural, autonomic, and plasticity-related mechanisms potentially relevant to recovery after severe brain injury. Nevertheless, differences in patient populations, stimulation protocols, comparators, and outcome measures, as well as the limited size of several studies, preclude direct extrapolation to disorders of consciousness. Evidence from these conditions should therefore be regarded as a translational rationale for investigating taVNS in DoC rather than as proof of efficacy in this population.

## 5. taVNS in Disorders of Consciousness

### 5.1. Translational Relevance for Disorders of Consciousness

The wide spectrum of clinical applications of taVNS provides important translational support for its use in disorders of consciousness. Across conditions such as depression, epilepsy, stroke, and chronic pain, taVNS has consistently demonstrated the ability to modulate brainstem neuromodulatory nuclei, thalamocortical circuits, and large-scale brain networks. These are the same systems critically disrupted in disorders of consciousness.

Furthermore, the favorable safety profile, non-invasive nature, and ease of administration make taVNS particularly suitable for medically fragile patients with severe brain injury. Evidence of enhanced neuroplasticity, autonomic stabilization, and anti-inflammatory effects across multiple clinical contexts further strengthens the biological plausibility of taVNS as a therapeutic strategy in disorders of consciousness.

### 5.2. Biological Mechanisms of taVNS in Disorders of Consciousness

Transcutaneous auricular vagus nerve stimulation (taVNS) is thought to exert its effects primarily through activation of vagal afferent fibers projecting to NTS, the main visceral sensory nucleus of the medulla oblongata [3,7]. The NTS represents a critical gateway through which peripheral interoceptive signals influence central autonomic, neuromodulatory, and arousal-related networks. From the NTS, second-order projections reach multiple brainstem nuclei that are key components of the ascending reticular activating system (ARAS), including the locus coeruleus (LC) and the dorsal raphe nuclei (DRN) [8,9,10].

Activation of the LC leads to widespread release of noradrenaline across the cortex, thalamus, and limbic structures, enhancing neuronal excitability, signal-to-noise ratio, and synaptic plasticity. The LC–noradrenergic system plays a central role in vigilance, attention, and arousal regulation, and its dysfunction has been implicated in disorders of consciousness [9]. In parallel, projections from the NTS to the DRN modulate serotonergic transmission, which is involved in mood regulation, behavioral activation, and cortical excitability [10]. Beyond brainstem neuromodulatory nuclei, NTS projections also reach the parabrachial nucleus, hypothalamus, thalamus, and basal forebrain [7,8]. These structures form part of a distributed network regulating wakefulness, autonomic control, and behavioral responsiveness. In particular, the thalamus plays a pivotal role in the generation and maintenance of conscious states through its reciprocal connections with the cortex. Impairment of thalamocortical loops is a hallmark of disorders of consciousness, leading to reduced cortical integration and impaired global neuronal workspace dynamics [2].

By modulating NTS-centered pathways and their downstream projections, taVNS may influence thalamocortical excitability and promote large-scale network integration. Indeed, through the coordinated activation of noradrenergic and serotonergic systems, taVNS may facilitate the re-engagement of cortical and subcortical circuits underlying conscious processing. Experimental and clinical studies have shown that vagus nerve stimulation can enhance cortical responsiveness, increase electroencephalographic complexity, and modulate event-related potentials such as the P300, which are considered electrophysiological markers of cognitive processing and awareness [15,30]. These findings support the hypothesis that vagal stimulation may facilitate the transition from a functionally disconnected state toward a more integrated and responsive brain network configuration.

Studies in other clinical conditions have shown that the vagus nerve also modulates autonomic balance and cardiovascular variability [27]. taVNS has been shown to increase parasympathetic tone and heart rate variability, reflecting enhanced vagal activity [26]. In patients with disorders of consciousness, autonomic dysregulation is common and has been associated with poorer outcomes. Improvements in autonomic responsiveness may therefore reflect a broader reorganization of central autonomic networks and could serve as indirect markers of recovery of consciousness.

At the synaptic and molecular level, vagal afferent stimulation is associated with increased release of neuromodulators such as noradrenaline, serotonin, and acetylcholine, as well as upregulation of neurotrophic factors including brain-derived neurotrophic factor (BDNF) [31]. These neurochemical changes facilitate synaptic plasticity, long-term potentiation, and network reorganization. In experimental models, vagus nerve stimulation paired with behavioral or sensory training enhances experience-dependent plasticity, supporting the concept of activity-dependent neuromodulation [32].

Finally, the anti-inflammatory effects of vagal activation may contribute indirectly to recovery processes. Through the cholinergic anti-inflammatory pathway, vagal efferent activity modulates cytokine release and systemic inflammation [12,13]. Given the role of neuroinflammation in secondary brain injury and chronic disorders of consciousness, modulation of inflammatory pathways may represent an additional mechanism supporting neural recovery.

This multi-level action supports the biological plausibility of taVNS as a neuromodulatory intervention in disorders of consciousness and aligns with contemporary network-based models of recovery.

#### Demonstrated Versus Proposed Mechanisms: Insights from Thalamic Stimulation Studies

The mechanistic account outlined above is largely extrapolated from studies conducted in healthy volunteers, animal models, and other neurological or psychiatric populations; direct causal evidence that vagal afferent stimulation modulates thalamocortical activity specifically in DoC patients themselves remains limited.

In this regard, studies employing direct electrical stimulation of thalamic nuclei in DoC patients provide complementary and more direct mechanistic support for a thalamic route to consciousness recovery. Deep brain stimulation (DBS) targeting central thalamic structures, particularly the centromedian–parafascicular (CM-Pf) complex, has produced measurable increases in behavioral responsiveness in patients with MCS and UWS/VS, ranging from an early single-case report [33] to larger case series and cohort studies [34,35,36,37]. These findings demonstrate that direct modulation of thalamic relay and associative nuclei can produce clinically detectable shifts along the consciousness spectrum, supporting mesocircuit-based models in which thalamocortical and thalamostriatal disruption underlies chronic unresponsiveness [38].

These findings offer valuable translational insight for taVNS. While taVNS is hypothesized to influence thalamocortical circuits indirectly, via NTS-mediated brainstem projections, this pathway has not yet been directly verified in DoC patients using invasive electrophysiology or targeted neuroimaging, unlike the thalamic DBS literature discussed above. The convergence between direct thalamic stimulation studies and the proposed thalamic target of taVNS strengthens the biological rationale for the latter, but also underscores that current evidence for taVNS’s central mechanism in DoC remains largely inferential.

### 5.3. The Clinical Role of taVNS in Disorders of Consciousness: Evidence from the Literature

Clinical research investigating taVNS in disorders of consciousness (DoC) is still in its early stages but has expanded considerably over the past decade. Initial evidence has primarily come from case reports and small pilot studies, followed more recently by controlled trials exploring behavioral and neurophysiological outcomes (Table 2).

One of the first reports suggesting a potential role for vagus nerve stimulation in DoC was the case study by Corazzol et al. [39], which described the recovery of behavioral signs of consciousness in a patient with chronic unresponsive wakefulness syndrome following invasive cervical vagus nerve stimulation. Although this intervention was invasive and involved a single patient, the study provided proof of concept that vagal neuromodulation could modulate large-scale brain networks associated with consciousness. Neuroimaging and electrophysiological findings in this case showed increased metabolic activity in thalamocortical regions and enhanced EEG connectivity, supporting the hypothesis that vagal stimulation may promote the reactivation of residual networks [39].

Subsequent case reports and case series focused on non-invasive auricular stimulation confirmed these promising results [40,41,42]. At the same time, hypotheses about the biological functioning of the method have encouraged the carrying out of RCTs. Early pilot investigations reported that taVNS could induce measurable changes in cortical activity and autonomic regulation. Despite these encouraging findings, the specific neural mechanisms underlying the effects of taVNS remained largely unclear, leaving a significant gap in the understanding of how this technique might influence brain function and, ultimately, clinical outcomes. Nevertheless, the consistency of these preliminary results across different populations pro-vided a positive rationale for further investigating the biological plausibility of taVNS, paving the way for more struc-tured mechanistic models and controlled clinical trials. Briand et al. [14] reviewed the emerging evidence and reported preliminary findings from fMRI findings of healthy subjects and proposed a vagal cortical pathways model explaining the influence of taVNS on brain activity. The 6-step model produces an increase in activity within the default mode network through the serotonin pathway, activation of the ascending reticular activating system and of the thalamus, and re-establishment of the cortico-striatal-thalamic-cortical loop.

taVNS was later longitudinally evaluated in a patient with prolonged disorders of consciousness [43] treated with repeated sessions over a long period of six months. The authors reported increases in Coma Recovery Scale–Revised (CRS-R) score along with changes in EEG complexity measures.

More structured clinical investigations have been conducted in recent years. A randomized controlled trial conducted by Zhou et al. [44] provided robust evidence for the clinical effects of taVNS. In this study, between November 2021 and October 2022, 60 patients with DoCs, including both MCS and VS/UWS, were randomized to active or sham taVNS. 57 patients completed the study (28 active, 29 sham. 32 MCS and 25 VS/UWS). The etiology was predominantly vascular and traumatic (stroke 60.71% vs. 44.83%; TBI 39.29% vs. 55.17% across the two arms). Time since the acute event was around four months (117.89 ± 15.91 days active vs. 124.31 ± 19.01 sham). Stimulation used a fixed intensity (level 15, 20 Hz, 200 µs pulse width, left ear, 30 min × 2/day, 6 days/week, 4 weeks), with a zero-current sham and conventional treatment in both arms; baseline CRS-R was 8.96 ± 4.02 vs. 8.72 ± 4.23. At the whole-group level, active taVNS showed improved consciousness versus sham, but this was not statistically significant. Only when the sample was split, the MCS subgroup showed a significant benefit, while the VS/UWS subgroup showed no significant difference between active and sham.

The intervention was well tolerated, and no significant side effects were reported.

In a subsequent single-center study conducted by Zhou et al. [45], 50 adult patients in a minimally conscious state (MCS) were enrolled between January and July 2023 and randomly assigned to receive either active taVNS (n = 25) or sham taVNS (n = 25); the protocol involved a 4-week treatment period followed by an 8-week follow-up. The etiology included cases of hypoxic-ischemic encephalopathy (HIE); the median time elapsed since the acute event was 48 days (29.5–86.5) in the active group and 34 days (26.5–57) in the sham group. Stimulation parameters were identical to those of the first study regarding frequency, duration, and location, but intensity was personalized (15–20, titrated using the *Nociception Coma Scale-Revised*) rather than fixed; the sham procedure was identical to that used in the first study by Zhou et al. [44]. Baseline CRS-R scores were 10 (9–11) in the active group and 9 (8–11) in the sham group. The active group showed significantly greater improvement in CRS-R and GCS scores compared to the sham group, reaching statistical significance between the third and fourth weeks; this improvement was accompanied by parallel progress in EEG and P300 parameters, as well as better scores on the Disability Rating Scale at 12 weeks.

The design of the second trial by Zhou et al. [45] suggests a direct methodological refinement compared to the first one [44], as the phenotype was limited to MCS and the sample size was calculated based on the effect size observed in the MCS subgroup of the first study; the intervention occurred earlier with personalized intensity. This would explain why only the second study achieved statistical significance for the CRS-R, a finding confirmed by convergent neurophysiological measures and a 12-week functional follow-up, elements that were absent in the first study.

A recent randomized study showed that the autonomic response (HRV) induced by taVNS can predict changes in the level of consciousness in DoC patients (MCS and UWS), suggesting a potential prognostic and therapeutic role [46]. It was a prospective clinical trial conducted from February 2022 to February 2024 at Hangzhou Armed Police Hospital, enrolling healthy controls and DoC patients, each given a single 10-min session of taVNS with ECG monitoring, The study used heart rate variability and a support vector machine classifier to see whether the acute autonomic reaction to one stimulation session could distinguish DoC patients from controls and predict later changes in consciousness level. Using HRV parameters, DoC and HC were correctly classified with an accuracy of 86%; MCS and UWS were classified with an accuracy of 78%.

## 6. Technical Aspects of taVNS

### 6.1. Invasive Versus Non-Invasive Vagus Nerve Stimulation

Across the revised literature, different vagus nerve stimulation (VNS) techniques have been investigated, broadly categorized into invasive and non-invasive approaches. These modalities differ substantially in their mechanisms of delivery, safety profiles, technical complexity, and clinical applicability. While invasive strategies rely on surgically implanted devices to achieve direct and sustained neuromodulation, non-invasive techniques aim to access vagal pathways transcutaneously, reducing procedural burden and associated risks. The following sections outline the main characteristics of these approaches, highlighting their methodological differences and clinical implications for disorders of consciousness.

Invasive vagus nerve stimulation (iVNS) involves surgical implantation of a cervical electrode and is an established therapy for drug-resistant epilepsy [17] and treatment-resistant depression [50]. Although iVNS provides robust neuromodulatory effects, it is associated with surgical risks, including infection, dysphonia, and cardiac complications. The exact mechanism by which iVNS achieves its effects is not known, but various mechanisms have been proposed, including afferent vagal projections to brain regions that generate epileptic seizures and the resulting desynchronization of cortical activity [17]. iVNS is implanted with a blunt technique on the left side to avoid cardiac side effects through the classic approach for anterior cervical discectomy [17]. This technique is approved by the FDA in the United States for the treatment of drug-resistant epilepsy and drug-resistant depression [51]. The device allows for the adjustment of parameters such as intensity, frequency, and pulse duration, with typical initial settings of 1.0–2.0 mA, 500 μs pulse width, 20–30 Hz, 30 s ON, and 5 min OFF [52].

In patients with disorders of consciousness, invasive VNS has been reported only in isolated cases. Corazzol et al. [39] described partial recovery of consciousness in a patient with chronic UWS following cervical VNS implantation, providing proof-of-concept evidence of vagal modulation in severe brain injury. However, ethical considerations, surgical burden, and medical fragility limit the applicability of invasive approaches in this population.

Transcutaneous auricular VNS is a non-invasive, reversible, and repeatable alternative with a favorable safety and tolerability profile [4,5]. taVNS uses electrodes applied to the skin, particularly on the ear (cymba conchae), where the auricular branch of the vagus nerve passes. This method does not require surgical intervention and can be self-administered by the patient. There are various protocols and devices, including respiratory-synchronized auricular stimulation (RAVANS), which integrates breathing with stimulation to enhance the activation of central circuits [53,54]. Recently, selective stimulation paradigms (sVNS) have been developed, which aim to modulate specific types of nerve fibers or anatomical areas of the nerve to reduce side effects and increase therapeutic specificity [55].

Across studies, stimulation protocols have been relatively consistent, typically targeting the cymba conchae with frequencies between 20 and 30 Hz, pulse widths of 200–500 microseconds, and intensities adjusted to individual tolerance (Table 3). Sessions are usually administered daily over several weeks (from 4 weeks up to 6 months [47].

Importantly, taVNS has demonstrated a favorable safety profile, with only minor local adverse effects such as skin irritation or discomfort reported.

Recent literature emphasizes that taVNS is generally well tolerated in patients with consciousness disorders, but recommends careful cardiac monitoring during the procedure, especially in subjects with cardiovascular comorbidities [56]. No additional absolute contraindications have emerged in clinical trials and systematic reviews, but patient selection must be rigorous to avoid predictable adverse events [57].

By preferentially activating vagal afferents projecting to the NTS, taVNS minimizes efferent stimulation and associated adverse effects. Consequently, current research trends favor taVNS as the preferred neuromodulation strategy in disorders of consciousness.

### 6.2. Technical Innovations in taVNS

Technological development in transcutaneous auricular vagus nerve stimulation (taVNS) has progressively focused on improving stimulation selectivity, safety, and reproducibility. Early devices were primarily adapted from general-purpose transcutaneous electrical stimulation systems and often employed non-specific electrode placements, such as the tragus or the earlobe, with limited anatomical precision. However, anatomical studies have demonstrated that the cymba conchae represents the auricular region most selectively innervated by the auricular branch of the vagus nerve, with minimal contribution from trigeminal or cervical sensory fibers [58]. These findings have driven the development of anatomically contoured electrodes specifically designed to target this region. Modern taVNS systems typically use flexible silicone-based electrodes or in-ear designs that adapt to the shape of the cymba conchae, improving contact stability and reducing impedance variability during repeated stimulation sessions. Functional neuroimaging studies have confirmed that stimulation at this site activates central vagal projections, including the nucleus tractus solitarius, locus coeruleus, and thalamic structures, supporting the rationale for anatomically guided electrode placement [59].

Another major technological improvement concerns the optimization of stimulation waveforms. Contemporary taVNS devices generally employ biphasic, charge-balanced pulses, which reduce the risk of skin irritation and prevent tissue damage during long-term use. This approach, which is standard in other neuromodulation techniques, has significantly improved the safety and tolerability profile of taVNS [5]. Although stimulation parameters vary across studies, most clinical protocols employ frequencies between 1 and 30 Hz, pulse widths ranging from 200 to 500 microseconds, and current intensities individually adjusted to sensory threshold or mild discomfort.

Recent technological advances have also introduced the concept of closed-loop or biomarker-guided stimulation. In these systems, stimulation parameters are dynamically adjusted based on physiological or neurophysiological signals, such as respiration, heart rate variability, or electroencephalographic activity. The rationale behind these approaches is to synchronize stimulation with endogenous neural rhythms, thereby enhancing plasticity and network responsiveness. Preliminary studies have suggested that respiration-synchronized taVNS may produce stronger autonomic and cortical effects than continuous stimulation, although these approaches remain largely experimental [16].

In parallel, the integration of taVNS with multimodal neurophysiological monitoring has become increasingly relevant, particularly in disorders of consciousness. Recent studies have combined taVNS with electroencephalography, event-related potentials, and autonomic measures to obtain objective markers of cortical reactivity and network integration [15,47,50]. Such approaches may help identify responders and non-responders and enable the development of personalized stimulation protocols. For example, clinical studies in patients with disorders of consciousness have reported changes in electroencephalographic complexity and event-related potentials following taVNS, suggesting engagement of residual cortical networks [15].

Finally, miniaturization and the development of portable or wearable taVNS systems have significantly improved the feasibility of long-term neuromodulation. Modern devices are typically battery-powered, programmable, and suitable for repeated daily sessions at the bedside or in outpatient settings. These technological advances have facilitated the translation of taVNS from experimental laboratory settings to real-world clinical environments, particularly in medically fragile populations such as patients with severe brain injury [8,9,10].

## 7. Discussion

This narrative review highlights transcutaneous auricular vagus nerve stimulation as a biologically plausible and clinically promising neuromodulation strategy for major clinical conditions, particularly disorders of consciousness. The rationale for taVNS is supported by converging anatomical, functional, biological, and physiological evidence implicating vagal afferent pathways and brainstem neuromodulatory nuclei in the regulation of arousal and thalamocortical connectivity [3,9,15].

Clinical evidence to date suggests that taVNS may preferentially benefit patients with various medical conditions, particularly those in a minimally conscious state. These findings are consistent with contemporary network-based models of consciousness, which emphasize residual network integrity and preserved large-scale connectivity as key determinants of responsiveness and recovery [1,2]. Preliminary clinical studies indeed suggest that taVNS is safe, feasible; although the efficacy of TAVNS remains to be determined with larger multicenter controlled trials, the intervention has been associated with improvements in behavioral responsiveness, electroencephalographic reactivity, and indices of cortical integration [39,40,41,42,43,44,45,46]. Moreover, the technique has shown a favorable safety profile, with only minor local adverse effects reported, making it particularly suitable for fragile populations with severe brain injury [15,16]. Furthermore, a major limitation in the analysis of these results is that the literature on DoC remains limited, and several studies originate from overlapping research groups, leading to a risk of publication bias.

Beyond its mechanistic and clinical implications, the relevance of taVNS must also be considered within the broader context of care for patients with DoC, a clinical population characterized by medical complexity, diagnostic uncertainty, and frequent marginalization within healthcare systems. These patients often require surrogate decision-making, raising substantial ethical and legal challenges, while limited specialized resources may restrict access to innovative interventions. In this setting, a non-invasive, well-tolerated, and mechanism-driven approach such as taVNS acquires particular value, not only as a potential therapeutic tool but also as a strategy that can be implemented with relatively low procedural burden.

Furthermore, technological innovations and biomarker-guided approaches further enhance the translational potential of taVNS into clinical practice, particularly when integrated with multimodal neurophysiological monitoring. From a methodological and technological standpoint, the use of standardized and reproducible taVNS protocols, in accordance with established guidelines, is essential to ensure both stimulation safety and comparability of results across studies. Accurate electrode placement, typically at the cymba conchae or tragus based on anatomical landmarks, together with appropriate parameter settings, is critical for effective vagal activation. Incorrect positioning or suboptimal stimulation parameters may lead to insufficient neuromodulatory effects or patient discomfort. At the same time, key parameters, including electrode location, current intensity, stimulation frequency, and session duration, may require individual adjustment or temporary discontinuation in the presence of adverse effects to maintain patient safety. If electrode contact is inadequate, repositioning the electrode or applying conductive gel may improve signal stability. Such troubleshooting strategies are important for preserving the reliability and safety of the stimulation protocol. In parallel, standardized diagnostic and prognostic assessments are necessary to evaluate the clinical impact of taVNS. Both behavioral scales and neurophysiological measures, such as electroencephalography, should be employed to detect changes in consciousness, including in the absence of overt behavioral responses. Assessments should be aligned with the patient’s circadian rhythms and fluctuating levels of arousal to reduce the risk of misdiagnosis. Finally, evaluations should be performed at baseline and during extended follow-up periods to determine the persistence and clinical relevance of treatment effects.

Despite these promising findings, several methodological limitations must be considered. Most studies involve small sample sizes, heterogeneous patient populations, and relatively short follow-up periods. Patients in a minimally conscious state (MCS) and those in unresponsive wakefulness syndrome/vegetative state (UWS/VS) do not constitute a homogeneous clinical group, but rather represent distinct entities, characterized by differing degrees of integrity of residual cortical and thalamocortical networks. The better response to taVNS observed in MCS patients may plausibly be explained by greater preservation of long-range functional connectivity, particularly within high-order networks and the thalamo-striato-cortical circuit, which likely represent the neural substrate required for vagal stimulation to translate into a measurable behavioral effect. This biological heterogeneity has a direct clinical implication: the distinction between MCS and UWS/VS based on behavioral assessment alone is burdened by a high rate of diagnostic error, even in specialized centers, due to fluctuations in arousal, concurrent motor impairment, and subtle or rapidly habituating behavioral responses [60]. It is therefore possible that part of the variability in taVNS response reported in the literature is partly attributable to imprecise diagnostic classification rather than a genuine difference in biological responsiveness, which reinforces the need to combine standardized behavioral assessment with neurophysiological and multimodal neuroimaging tools for more accurate diagnostic stratification.

A further, related source of heterogeneity is the etiology of the brain injury underlying DoC. Traumatic, anoxic-ischemic, and vascular forms are not biologically equivalent: the diffuse axonal injury typical of traumatic brain injury produces a multifocal lesion pattern that often partially spares thalamocortical connections, whereas global anoxic-ischemic injury tends to affect subcortical structures and the cortex more diffusely and symmetrically; vascular lesions, in turn, produce more focal patterns that depend on the arterial territory involved. These differences translate into substantially different patterns of residual connectivity and recovery trajectories. It is therefore crucial that future studies evaluate potential differential responses to taVNS based on the diverse etiologies of disorders of consciousness (DoC). Spontaneous recovery and fluctuations in responsiveness are also possible; consequently, the interpretation of results remains limited, particularly in light of the clinical course of disorders of consciousness (DoC), which based on current knowledge cannot be predicted with certainty.

Variability in stimulation parameters, session duration, and outcome measures further complicates comparisons across studies. Most studies are exploratory or pilot in nature, and only a few randomized controlled trials have been conducted to date. As a result, the optimal stimulation parameters, treatment duration, and patient selection criteria have not yet been clearly defined [15]. In particular, the differential responsiveness observed between patients in minimally conscious state and those in unresponsive wakefulness syndrome highlights the need for better stratification based on neurophysiological and neuroimaging markers of residual network integrity. Finally, spontaneous recovery and fluctuations in responsiveness are also possible; consequently, the interpretation of results remains limited, particularly in light of the clinical course of disorders of consciousness (DoC), which, based on current knowledge, cannot be predicted with certainty.

A further limitation concerns the outcome measures used across the available taVNS studies in DoC, which rely mainly on aggregate behavioral scale scores or categorical diagnostic shifts, without demonstrating whether these changes translate into a genuine improvement in patients’ quality of life or a reduction in caregiver burden. This also raises an ethical question: an increase in indices of awareness is not inherently beneficial if it is not accompanied by a real improvement in communication, autonomy, and environmental interaction.

Future research should focus on large, multicenter randomized controlled trials using standardized stimulation protocols and multimodal outcome measures; longer follow-up periods are needed to establish its clinical effectiveness and identify optimal stimulation parameters. The integration of behavioral scales with neurophysiological and autonomic biomarkers may help identify responders, guide individualized stimulation strategies, and clarify the mechanisms underlying clinical improvement. In addition, technological innovations, including closed-loop and biomarker-guided stimulation systems, may enhance the precision and effectiveness of taVNS interventions [16].

At present, taVNS is not recommended as a standard treatment by any major international scientific society. However, it is regarded as a promising neuromodulatory strategy that is still undergoing clinical validation. Current guidelines for disorders of consciousness emphasize established diagnostic and rehabilitative approaches, while neuromodulation techniques such as taVNS remain investigational and require further high-quality evidence before routine clinical adoption [61]. While early results are encouraging, robust clinical evidence is still needed to establish its therapeutic efficacy, define optimal treatment parameters, and identify the patients most likely to benefit from this intervention.

## 8. Conclusions

Transcutaneous auricular vagus nerve stimulation has emerged as a promising non-invasive neuromodulatory approach for patients with disorders of consciousness. The anatomical and physiological organization of the vagal afferent system provides a strong neurobiological rationale for its use, as stimulation of the auricular branch of the vagus nerve allows indirect modulation of brainstem neuromodulatory nuclei and thalamocortical circuits involved in arousal and conscious processing. This mechanism is consistent with contemporary network-based models of consciousness, which emphasize the role of large-scale connectivity and neuromodulatory tone in the recovery of awareness.

## 9. Future Directions

Beyond methodological challenges, ethical and organizational aspects must also be addressed. Patients with disorders of consciousness represent a highly vulnerable population, often dependent on surrogate decision-makers and subject to significant variability in access to specialized care. The development of shared clinical and research protocols, as well as interdisciplinary collaboration across neurology, rehabilitation, neurophysiology, and bioethics, will be essential to ensure equitable and scientifically sound implementation of taVNS.

## Figures and Tables

**Table 1:** Proposed clinical applications of transcutaneous auricular vagus nerve stimulation (taVNS).

Clinical Condition	Key Studies	Study Type	Proposed Main Targets	Reported Clinical Effects	Main Limitations
Drug-resistant epilepsy	Rong 2014 [18]; Bauer 2016 [19]	RCTs	Brainstem–thalamocortical circuits	Reduced seizure frequency, improved QoL and EEG	Smaller effect vs. iVNS
Major depressive disorder	Moeller 2019 [20]; Hein 2013 [21]; Rong 2016 [22]	Randomized and non-randomized controlled pilot study	Limbic–prefrontal networks	Reduction in depressive symptoms	Placebo effects
Migraine/headache	Straube 2015 [24]	RCT	Trigeminovascular system	Reduced attack frequency and intensity	Short follow-up
Chronic pain	Various pilot studies, Zang 2026 [25]	Pilot trials	Descending pain pathways	Pain reduction	Small samples
Autonomic dysfunction	Clancy 2014 [26]; Stavrakis 2015 [27]	Physiological and clinical studies	Parasympathetic pathways	Increased HRV, reduced sympathetic tone	Surrogate endpoints
Stroke rehabilitation	Wang 2024 [28]; Redgrave 2018 [29]	Controlled trials	Motor cortex plasticity	Improved motor recovery	Small samples

**Table 2:** Applications of transcutaneous auricular vagus nerve stimulation in disorders of consciousness.

Study	Population	Etiology/Time Since Injury	Study Design	Duration/Intervention	Main Outcomes	Safety
Corazzol et al., 2017 [39]	Single patient with chronic post-traumatic UWS	Traumatic (car accident); 15 years	Case report; invasive cervical VNS	Implanted VNS with long-term follow-up (several months)	Emergence of behavioral signs of consciousness; Increased thalamocortical metabolism and EEG connectivity	No adverse events
Yu et al., 2017 [40]	Single patients with UWS	Anoxic (cardiopulmonary arrest); 50 days	Case report	taVNS (twice daily for 30 min each in four consecutive weeks)	Transition to MCS; functional connectivity (FC) changes of the default mode network (DMN) at fMRI	Well tolerated
Noè et al., 2019 [41]	14 Patients with MCS-, VS, UWS	Traumatic: 7; Anoxic: 4; Hemorrhagic: 3; 12.1 ± 6.4 months	Prospective observational study	Twice a day (five days per week), 4 weeks follow up	Five of the eight MCS patients at admission showed an improvement in the CRS-R during the duration of the study	Well tolerated
Hakon et al., 2020 [42]	5 Patients with Diffuse Axonal Injury	Traumatic (diffuse axonal injury), all 5 pts; median 41 days (range 31–95)	Case series	4 h daily for eight weeks	Three patients showed improvements (>3 points) in the CRS-R	Well tolerated
Osińska et al., 2022 [43]	Single Patient with UWS	Traumatic; 6 years	Longitudinal case study	100 taVNS sessions (A single taVNS session lasted about 4 h)	Increase in CRS-R scores and EEG complexity (re-emergence of a second oscillatory peak in the alpha range)	Well tolerated; no major side effects
Zhou et al., 2023 [44]	57 Patients with DoC (MCS, UWS)	Stroke 30/57 (53%); TBI 27/57 (47%); mean ≈ 121 days (≈4 months)	Randomized double blind controlled trial (28 active taVNS, 29 sham taVNS)	30 min twice daily 6 days per week for 4 weeks; pulse width, 200 us; frequency, 20 Hz; intensity, 15	CRS-R greater improvement in active group (not statistically significant) Better results in MCS	No significant adverse event
Zhou et al., 2023 [45]	50 Patients with MCS	TBI 22/50 (44%); Non-TBI 28/50 (56%: stroke 24, HIE 4); median ≈ 40 days (range 26.5–86.5)	Randomized, sham-controlled trial (25 taVNS, 25 sham treatment)	30 min, twice daily, 6 days per week, over a period of 4 week	Greater improvement in CRS-R/GCS in patients receiving active stimulation	No significant adverse events
Li et al., 2025 [46]	Patients with DoC (17 MCS; 19 UWS)	Mixed acquired brain injury (breakdown not reported)	Prospective randomized clinical trial	10 min taVNS sessions with autonomic monitoring (total duration of the treatment not reported)	Autonomic responses (e.g., HRV changes) predicted improvement in level of consciousness	Well tolerated; no serious adverse events reported
Ongoing trials						
Zhou et al., 2024 [47] ChiCTR2300073950	Patients with prolonged DoC (multicenter); ongoing, actively recruiting	Mixed etiology (not restricted); pDoC, onset > 28 days	Multicenter, triple-blind, randomized controlled trial (study protocol; ongoing)	Repeated taVNS sessions 60 min two times per day for 4 weeks	Primary outcome: change in CRS-R scores; secondary outcomes: neurophysiological and functional measures	Safety to be evaluated; protocol reports expected good tolerability based on prior taVNS studies
Cheng et al., 2023 [48] ChiCTR2100045161	90 Patients with chronic DoC; intervention phase concluded	Mixed acquired brain injury (not restricted); onset > 28 days	Randomized controlled trial (sham taVNS or active taVNS)	Daily taVNS sessions 40 min per day, 5 days per week over a 40-day cycle	Primary outcomes: changes in standardized consciousness scales (CRS-R), Secondary outcomes: MRI, EEG, Phosphorylated tau (P-tau), and Neurofilament light chain (NFL)	
Jiao et al., 2025 [49] ITMCTR2024000734	50 patients with prolonged DOC; no explicit recruitment-status statement	Mixed acquired brain injury (not restricted); pDoC, onset > 28 days	Prospective, exploratory clinical trial	2 sessions of 30 min per day, 5 days per week, for a total period of 4 weeks	CRS-R, EEG parameters	

**Table 3:** Summary of stimulation parameters used across taVNS studies in disorders of consciousness.

Study	Stimulation Site	Frequency	Pulse Width	Intensity	Session Duration	Treatment Period
Corazzol et al., 2017 [39]	Cervical vagus nerve (surgically implanted, left side)	30 Hz	500 μs	0.25 mA initially, gradually increased to 1.5 mA	30 s ON/5 min OFF (duty cycle)	6 months
Yu et al., 2017 [40]	Cymba conchae (bilateral)	20 Hz	<1000 μs	4–6 mA	30 min, twice daily	4 consecutive weeks
Noè et al., 2019 [41]	Left tragus	20 Hz	250 μs	1.5 mA	30 min, twice daily, 5 days/week	4 weeks + 4-week follow-up
Hakon et al., 2020 [42]	Cymba conchae	25 Hz	250 μs	0.5 mA (first 3 days), then 1 mA	4 h daily (30 s ON/30 s OFF)	8 weeks
Osińska et al., 2022 [43]	Cymba conchae (left ear)	25 Hz	250 μs (0.25 ms)	0.2–1.5 mA (+0.1 mA/week)	4 h/day (continuous, 30 s ON/30 s OFF)	6 months (100+ sessions)
Zhou et al., 2023 [44]	Left outer ear (auricular branch)	20 Hz	200 μs	Gear 15 (device level)	30 min, twice daily, 6 days/week	4 weeks
Zhou et al., 2023 [45]	Cymba conchae (left ear)	20 Hz	200 μs	Gear 15–20, titrated via NCS-R	30 min, twice daily, 6 days/week	4 weeks + 8-week follow-up
Li et al., 2025 [46]	Cymba conchae	Alternating 4/20 Hz (3 s/7 s cycles)	200 μs	NR	Single 10-min session	Single-session study (no multi-week treatment)
Ongoing trials						
Zhou et al., 2024 [47]	NR (auricular)	25 Hz	300 μs	1 mA	60 min, twice daily (30 s ON/30 s OFF)	4 weeks + 4-week follow-up
Cheng et al., 2023 [48]	Cymba conchae and inner tragus (left, or left + right)	Alternating 20 Hz (7 s)/4 Hz (3 s) cycles	200 μs	NR (individually titrated)	40 min/day, 5 days/week	40-day cycle
Jiao et al., 2025 [49]	Cymba conchae and cavum conchae (bilateral)	Dense-sparse wave, 4/20 Hz	NR	~1–1.5 mA	30 min, 2 sessions/day, 5 days/week	4 weeks

## Data Availability

No new data were created or analyzed in this study. Data sharing is not applicable to this article.

## Abbreviations

The following abbreviations are used in this manuscript:

ABVN	Auricular branch of the vagus nerve
AP	Area postrema
ARAS	Ascending reticular activating system
BAEP	Brainstem auditory evoked potential
BDNF	Brain-derived neurotrophic factor
CRS-R	Coma recovery scale–revised
DMN	Default mode network
DMNV	Dorsal motor nucleus of the vagus
DoC	Disorders of consciousness
DRN	Dorsal raphe nuclei/nucleus
EEG	Electroencephalography/electroencephalographic
FC	Functional connectivity
FDA	Food and drug administration
fMRI	Functional magnetic resonance imaging
GCS	Glasgow coma scale
HC	Healthy controls
HRV	Heart rate variability
IL-1β/IL-6	Interleukin-1 beta/interleukin-6 (interleuchina-1 beta/interleuchin-6)
iVNS	Invasive vagus nerve stimulation
LC	Locus coeruleus
MCS	Minimally conscious state
NFL	Neurofilament light chain
NTS	Nucleus tractus solitarius
PAG	Periaqueductal gray
PB	Parabrachial nucleus
PNS	Parasympathetic nervous system
P-tau	Phosphorylated tau
QoL	Quality of life
RAVANS	Respiratory-gated/synchronized auricular vagal (afferent) nerve stimulation
RCT	Randomized controlled trial
SuS	Superior salivatory nucleus
SVM	Support vector machine
sVNS	Selective vagus nerve stimulation
TAVREC	Transcutaneous auricular vagal nerve stimulation for consciousness recovery
TCC	Trigeminocervical complex
TNF	Tumor necrosis factor
UWS/VS	Unresponsive wakefulness syndrome/vegetative state
VNS	Vagus nerve stimulation
taVNS	Transcutaneous auricular vagus nerve stimulation
